# Comparative Performance of Patch-Type and Lead-Type Wearable Electrocardiogram Devices for Arrhythmia Detection in Routine Clinical Practice

**DOI:** 10.3390/jcm15020526

**Published:** 2026-01-08

**Authors:** Dong Geum Shin, Bokyoung Kim, Jihyun Ahn, Hyung Wook Park, Namsik Yoon, Kihong Lee, Yoo Ri Kim

**Affiliations:** 1 Division of Cardiology, Hallym University Medical Center, Kangnam Sacred Heart Hospital, Seoul 07441, Republic of Korea; blaudg@naver.com; 2 Research Institute of Nursing Innovation, College of Nursing, Kyungpook National University, Daegu 41566, Republic of Korea; bonnie@knu.ac.kr; 3 Department of Internal Medicine, Korea Medical Institute, Seoul 04522, Republic of Korea; jihyunahnmd@gmail.com; 4 Division of Cardiology, Department of Internal Medicine, College of Medicine, Chonnam National University, 42 Jebong-Ro, Dong-Gu, Gwangju 61469, Republic of Korea; mdhwp@chol.com (H.W.P.); nsaids77@chonnam.ac.kr (N.Y.); drgood2@jnu.ac.kr (K.L.)

**Keywords:** arrhythmias, atrial fibrillation, electrocardiography, wearable electronic devices

## Abstract

**Background/Objectives:** Wearable electrocardiogram (ECG) monitoring has become increasingly important in detecting atrial fibrillation (AF) and subclinical arrhythmias by addressing diagnostic gaps inherent to intermittent or asymptomatic presentations. In contemporary clinical practice, two major types of wearable ECG monitors—patch-type and lead-type—are widely used, each with distinct advantages and limitations. This study aims to compare these modalities and evaluate their respective strengths and constraints in real-world settings. **Methods:** We retrospectively analyzed 639 consecutive outpatients (mean age 61.7 ± 14.5 years; 56.7% male) who underwent wearable ECG monitoring between March 2022 and October 2023. Patients were stratified into patch-type (n = 466; 72.9%) and lead-type (n = 173; 27.1%) groups. Baseline characteristics were comparable. Indications, monitoring duration, arrhythmia detection, and noise rates were assessed. **Results:** Baseline characteristics did not differ significantly between the two groups. Lead-type monitoring was often prescribed for symptomatic patients (87.9% vs. 75.8%; *p* = 0.001), Symptomatic patients were older than asymptomatic patients (*p* = 0.040), whereas the proportion of males was higher in the asymptomatic group (*p* < 0.001). AF detection rates were comparable between the two groups (24.0% vs. 24.9%; *p* = 0.911). Patch-type monitoring achieved significantly longer recording duration (*p* < 0.001) and higher pause event detection (*p* = 0.004), but at the cost of increased noise burden (*p* < 0.001). **Conclusions:** Both patch-type and lead-type wearable ECGs are clinical applicable for arrhythmia surveillance in real-world practice. While AF detection rates were similar, the patch-type monitoring provided more extended observation periods and enhanced pause detection, though accompanied by a higher noise burden. These findings suggest that device selection should be individualized based on patient symptoms, monitoring goals, and tolerability. This study provides practical insights for optimizing wearable ECG use in routine practice.

## 1. Introduction

Cardiac arrhythmias remain a leading cause of morbidity and mortality worldwide, contributing to stroke, heart failure (HF), and sudden cardiac death. Timely and accurate diagnosis is essential for improving patient outcomes; however, conventional modalities such as 12-lead electrocardiography and 24–48 h Holter monitoring are often insufficient for detecting intermittent or paroxysmal arrhythmias. These limitations have driven interest in extended, patient-friendly rhythm monitoring solutions [1].

Advances in sensors, connectivity, and digital platforms have enabled long-term ambulatory electrocardiogram (ECG) monitoring beyond the hospital setting, democratizing access to cardiac care [2]. Studies on smartwatch-based atrial fibrillation (AF) detection have highlighted the clinical value of wearable ECG modalities in enhancing arrhythmia detection and supporting appropriate patient care [3,4]. In parallel, international societies emphasize that mobile health (mHealth) tools—including wearable ECG patches and smartphone-based applications—represent a natural evolution of remote monitoring, potential to extend surveillance from high-risk patients to broader populations [5,6,7].

Clinical studies have reinforced the diagnostic advantages of wearable ECG systems. Wearable ECG devices have shown superior sensitivity for detecting paroxysmal AF compared with Holter monitors, particularly in patients with cryptogenic stroke or unexplained palpitations [8]. Moreover, innovative wearable platforms increasingly integrate automated arrhythmia detection into daily life, allowing real-time event capture without disrupting routine activities. Such remote cardiac rhythm monitoring has demonstrated improved diagnostic yield and higher patient adherence and satisfaction compared with conventional modalities [9].

Despite these advances, several challenges remain regarding validation, integration into electronic medical records, regulatory oversight, and reimbursement models. Concerns regarding false positive detection, clinical workflow burden, and equitable access underscore the need for careful evaluation before widespread implementation [2,5]. Therefore, systematic comparisons of different wearable ECG modalities are essential to guide evidence-based implementation and to clarify their role in evolving arrhythmia management strategies.

Most studies on single-ECG devices, a type of wearable ECG devices, have been conducted as comparisons with conventional monitoring methods [8,10,11,12]. Currently, single-lead ECG devices used in routine clinical use can be broadly categorized into lead-type and patch-type devices; however, no studies have directly compared these two types. Although both patch-type and lead-type wearable ECG devices are widely used for single-lead ambulatory rhythm monitoring, direct head-to-head comparisons between these two device categories are limited. In routine clinical practice, clinicians are often required to choose between patch-type and lead-type devices based not only on diagnostic objectives but also on practical considerations such as expected monitoring duration, patient symptoms, tolerability, and signal quality. However, the absence of comparative real-world data makes evidence-based device selection challenging. A direct comparison between patch-type and lead-type wearable ECG devices is therefore necessary to clarify their relative strengths and limitations and to inform individualized device selection in clinical settings.

In this study, we aimed to compare the diagnostic performance and operational characteristics of commercially available single-lead wearable ECG devices, including one patch-type device and two lead-type devices, used in routine clinical practice, focusing on AF detection, monitoring duration, pause event identification, and noise burden.

## 2. Methods

This retrospective observational study consecutively enrolled patients who underwent wearable ECG monitoring at the outpatient clinics of two tertiary referral centers between March 2022 and October 2023. A total of 639 patients comprised the study cohort. Baseline demographics and clinical characteristics were extracted from medical records, including comorbidities such as hypertension, diabetes mellitus, dyslipidemia, coronary artery disease (CAD), HF, prior stroke/transient ischemic attack (TIA), and chronic kidney disease (CKD). Disease definitions were based on the World Health Organization International Classification of Diseases (ICD), and diagnoses were classified as present when the corresponding ICD codes were recorded.

The study protocol was reviewed and approved by the Institutional Review Board (IRB) and the requirement for informed consent was waived due to the retrospective study design (IRB No. CNUH-2023-167; Big data construction of patients with cardiac arrhythmia).

Patients were stratified into two groups based on the electrocardiographic monitoring modality: patch-type wearable ECG (n = 466) and lead-type wearable ECG (n = 173). Device selection not randomized but was determined by the treating physician, taking into account clinical indications and patient characteristics.

Patch-type monitoring was performed using a single commercially available wearable ECG device (AT-Patch, ATsens, Seongnam-si, Republic of Korea). Lead-type monitoring included two commercially available wearable ECG devices (mobiCARE, Pyeongtaek-si, Republic of Korea, SEER, Pyeongtaek-si, Republic of Korea, and S-Patch, Wellysis, Seoul, Republic of Korea), which share a similar single-lead configuration and operating principles. Because these lead-type devices have comparable signal acquisition methods and clinical indications, they were analyzed together as a single lead-type category. Although multiple wearable ECG devices were included, the primary comparison in this study was based on device category (patch-type versus lead-type), reflecting differences in form factor and clinical use rather than manufacturer-specific performance. The patch-type device and the two lead-type devices used in this study shared comparable single-lead ECG configurations, with a sampling rate of 250~256 Hz and a clinical ECG frequency bandwidth of approximately 0.05–55 Hz, which are sufficient for AF detection in routine clinical practice. A comparison of the key technical features of the patch-type and lead-type wearable ECG devices used in this study is summarized in Supplementary Table S1. Noise rate was assessed using the device-specific automated signal quality algorithms embedded in each monitoring system. For both patch-type and lead-type wearable ECG devices, noise was defined as ECG segments in which reliable QRS detection was not possible because of signal artifacts. Noise detection was performed using automated algorithms based on RR-interval irregularity and signal morphology, which identify segments with excessive signal distortion, motion artifacts, or electrode detachment. The mean noise rate was calculated as the proportion of total recording time classified as noise relative to the entire monitoring duration and was expressed as a percentage.

The primary endpoint was the detection of AF. Secondary endpoints included total monitoring duration, QRS complex counts, incidence of pause events, and noise percentage. Monitoring indications were classified as either symptom-driven (palpitations, dizziness, syncope, or chest discomfort) or non-symptom-driven (arrhythmia follow-up or evaluation of abnormal ECG findings).

Continuous variables were reported as mean ± standard deviation and analyzed using the Student *t*-test or the Mann–Whitney U test as appropriate. The normality was determined using the Kolmogorov–Smirnov goodness-of-fit test. Categorical variables were reported as counts and proportions, and were analyzed using Pearson’s chi-square tests or Fisher’s exact tests, as appropriate. A *p* value ≤ 0.05 was considered statistically significant. Standardized effect sizes for between-group comparisons of continuous variables were calculated using Cohen’s d, with Hedges’ g applied as a small-sample correction. All analyses were conducted according to pre-specified protocols on complete datasets with no missing values. Statistical analyses were performed using SPSS Statistics for Windows, version 26.0 (IBM Corp., Armonk, NY, USA).

## 3. Results

### 3.1. Baseline Characteristics

**Figure 1:**
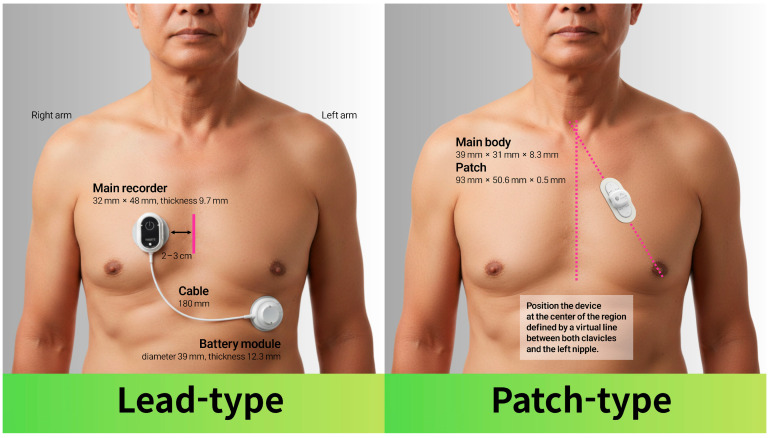
Examples of representative wearable electrocardiogram devices by category.

A total of 639 outpatients were enrolled in the study, with a mean age of 61.7 ± 14.5 years and a male predominance of 56.7%. Of these patients, 466 (72.9%) underwent patch-type wearable ECG monitoring and 173 (27.1%) underwent lead-type wearable ECG monitoring. Figure 1 illustrates the representative configurations and recommended placement of the two wearable ECG monitoring devices used in this study. The lead-type device consists of a main recorder connected via a short cable to a separate battery module, with electrodes positioned on the anterior chest approximately 2–3 cm apart. In contrast, the patch-type device is a single, integrated unit applied directly to the left anterior chest wall; it is positioned at the center of the region defined by a virtual line connecting both clavicles and the left nipple. Baseline characteristics and comorbidities were well balanced between the two groups. Specifically, there were no significant differences in patient age, sex, hypertension, diabetes mellitus, dyslipidemia, CAD, HF, prior stroke/TIA, and CKD (Table 1).

### 3.2. Indications for Monitoring

Symptom-driven indications represented the majority of cases (79.0%) (Table 2). Lead-type devices were more frequently prescribed for symptomatic patients compared with patch-type devices (87.9% vs. 75.8%, *p* = 0.001). Among symptomatic presentations, palpitations were the most common complaint (47.5%), followed by dizziness (20.0%), syncope (15.8%), and chest discomfort (9.7%). In contrast, non-symptom-driven indications, including arrhythmia surveillance and evaluation of abnormal ECG findings, were predominantly managed with patch-type devices.

Table 3 compares the baseline clinical characteristics between patients without symptoms and those with symptoms. In the overall cohort, symptomatic patients were significantly older than asymptomatic patients (62.3 ± 14.9 vs. 59.6 ± 12.8 years, *p* = 0.040), while the proportion of males was higher in the asymptomatic group (72.4% vs. 52.5%, *p* < 0.001). Although hypertension and dyslipidemia differed significantly between the two groups, no significant differences were observed for HF, diabetes mellitus, prior stroke/TIA, CAD, or CKD. Patient characteristics were classified according to symptoms within each device group (Supplementary Table S2). The lead-type device patients showed no significant differences in baseline characteristics between symptomatic and asymptomatic groups. In the patch-type group, symptomatic patients were older and more frequently female than asymptomatic patients. Hypertension and dyslipidemia were more prevalent among symptomatic patients in the patch-type group, while no significant differences were observed for other comorbidities.

### 3.3. Monitoring Parameters and Arrhythmia Detection

Patch-type devices provided significantly longer mean monitoring durations compared with lead-type devices (167.2 [144.9–168.7] h vs. 72.0 [71.7–72.0] h, *p* < 0.001) (Table 4). Consequently, the total QRS complex count recorded was higher in the patch-type group. The incidence of pause events lasting ≥2 s was also significantly greater with patch-type monitoring (72.5 [7.0–777.0] vs. 7.0 [2.0–53.0], *p* = 0.002). The AF detection rate was comparable between the two modalities (24.0% with patch-type vs. 24.9% with lead-type, *p* = 0.911). However, patch-type monitoring demonstrated a higher mean noise rate compared with lead-type monitoring (2.6% [0.9–6.7] vs. 7.9% [3.8–15.0], *p* < 0.001). In addition to *p*-values, standardized effect sizes were calculated to quantify the magnitude of between-group differences. Monitoring duration showed a very large effect size (Cohen’s d = 3.97), reflecting the inherent difference in prescribed monitoring duration between device types, whereas total QRS count and noise rate demonstrated moderate effect sizes (d = 0.70 and 0.43, respectively), and pause event counts showed a small effect size (d = 0.17).

## 4. Discussion

This study evaluated the diagnostic performance and clinical utility of patch-type and lead-type wearable ECG monitoring systems. A comparison of patch and lead-type wearable ECG monitoring showed that both methods were clinically feasible. Patch-type monitoring was used more frequently in asymptomatic patients. Despite similar atrial fibrillation detection rates, the patch-type demonstrated prolonged monitoring and increased pause events. However, this advantage was counterbalanced by a higher noise rate. The choice between these methods should be tailored to patient needs, considering monitoring duration, diagnostic accuracy, and noise tolerance. These findings support an individualized device selection strategy that incorporates clinical presentation (such as symptom severity), monitoring objectives (extended surveillance versus signal fidelity), and patient tolerance for noise artifacts.

The rapid expansion of mHealth technologies has provided a foundation for consumer- and clinician-facing wearable devices that enable continuous rhythm assessment. Technological advances continue to enhance the diagnostic capacity of wearable ECG systems. Artificial intelligence has improved noise reduction and arrhythmia classification, enabling accurate analysis in low-power, real-world environments [13]. Additionally, multimodal devices that integrate ECG and phonocardiography represent an innovative approach, enhancing arrhythmia detection through combined assessment of electrical and mechanical cardiac activity [14]. Recently, wearable ECG technologies have become an important innovation in arrhythmia detection and long-term cardiac rhythm monitoring. By extending the diagnostic window beyond the limitations of conventional Holter monitoring, these devices have demonstrated superior diagnostic yield, particularly for paroxysmal arrhythmias such as AF [8,15]. Clinical evidence indicates that patch-based and remote ECG solutions enhance patient adherence, comfort, and real-world applicability, positioning them as practical alternatives to short-term, clinic-based monitoring systems [16,17].

Clinical studies have consistently demonstrated the diagnostic advantages of patch-based monitoring compared with traditional Holter devices. Large-scale analyses have shown that patch-based monitoring provides superior sensitivity for detecting paroxysmal arrhythmias relative to Holter devices, with a substantial proportion of AF episodes detected beyond the initial 24-h recording period [8,12]. These findings underscore the clinical value of patch devices’ extended recording capability and user-friendly design. Also, head-to-head evaluations comparing wearable patches with conventional telemetry systems have also supported their diagnostic reliability. In hospitalized patients, patch-based monitoring demonstrated diagnostic accuracy comparable to continuous telemonitoring while providing advantages in patient mobility and reduced signal interruption [10]. Similarly, postoperative cardiac surgery patients monitored with wearable patches achieved arrhythmia detection rates comparable to Holter monitoring, confirming the feasibility of these devices in high-risk clinical settings [11]. Although Holter monitoring remains a well-established reference standard for short-term rhythm assessment, its limited recording duration restricts sensitivity for detecting paroxysmal arrhythmias. Wearable ECG devices address this limitation by enabling extended continuous surveillance in ambulatory settings. Conversely, Holter monitoring may still be advantageous when high signal fidelity over a short period is required, such as in patients with frequent symptoms or when precise arrhythmia characterization is necessary. Thus, wearable ECG devices should be viewed as complementary rather than substitutive to Holter monitoring, with device selection guided by clinical context and diagnostic objectives.

A variety of wearable ECG devices are currently available, and among them, single-lead, patch-based ECG monitors are being adopted rapidly in clinical practice. Clinically used single-lead wearable devices can broadly be classified into two categories—lead-type and patch-type—based on their form factor. Some hospitals use both types, whereas others use on a single device type. Although each device has distinct advantages and considerations regarding device application, patient comfort, and diagnostic accuracy, no study to date has directly compared these two devices. In this study, we compared the diagnostic performance and operational characteristics of lead-type and patch-type devices. The imbalance in sample size between the patch-type and lead-type groups warrants consideration when interpreting the study findings. This imbalance was not driven by the study design but rather reflects real-world clinical practice, in which patch-type devices are more frequently prescribed because of their longer battery life and suitability for prolonged monitoring, particularly in patients with infrequent or asymptomatic arrhythmias. As a result, this imbalance may have influenced secondary outcomes such as total monitoring duration, pause event detection, and noise burden, all of which are closely related to recording length and device characteristics. Importantly, however, the primary outcome of atrial fibrillation detection was comparable between the two groups, suggesting that the main conclusions of the study are robust despite the unequal group sizes.

An important technical distinction between the devices evaluated in this study relates to battery capacity and expected monitoring duration. The lead-type devices used in this study typically support approximately 3 days of continuous recording, whereas the patch-type device allows prolonged monitoring for up to 14 days without battery replacement. This difference in battery longevity largely explains the significantly longer monitoring duration observed in the patch-type group and the higher number of detected pause events. However, extended continuous recording may also increase susceptibility to motion- or adhesion-related artifacts, which could contribute to the higher noise burden observed with patch-type monitoring. Furthermore, the noise burden may be attributable to device attachment characteristics. Lead-type devices are not waterproof and therefore require removal during showering, but they allow reattachment if electrodes become detached. In contrast, patch-type devices are waterproof and permit continuous wear during daily activities, including showering; however, once partial detachment occurs, reattachment is generally not recommended. As a result, minor detachment during routine activities may have been recorded as noise, contributing to the higher noise rate observed in the patch-type group. Together, these findings highlight an inherent trade-off between prolonged monitoring capability and signal stability when selecting wearable ECG devices in routine clinical practice. The strengths of this analysis include the contemporary real-world outpatient cohort, direct comparison of two commonly utilized modalities, and comprehensive evaluation of operational metrics, including monitoring duration, noise burden, and pause event frequency.

The concept of individualized device selection is particularly relevant in the context of wearable ECG monitoring, where multiple device options with distinct technical characteristics are available. Our findings suggest that device choice should be guided by an integrated consideration of clinical presentation, monitoring objectives, and tolerance for signal artifacts. For patients with frequent or symptomatic events requiring high signal fidelity and immediate symptom–rhythm correlation, lead-type devices may be preferable because of their lower noise burden and ease of electrode reattachment. On the other hand, for patients with infrequent or asymptomatic arrhythmias, patch-type devices may offer advantages by enabling prolonged continuous monitoring, despite a higher susceptibility to noise artifacts. Importantly, the comparable AF detection rates observed between device types in this study suggest that extended monitoring duration may compensate for reduced signal fidelity in certain clinical scenarios.

Beyond diagnostic accuracy, successful implementation of wearable monitoring requires integration into clinical workflows and alignment with patient-centered outcomes. The mobile health field increasingly emphasizes arrhythmia detection, usability, adherence, and quality of life. Consensus recommendations from major medical societies underscore the importance of validated protocols, electronic health record interoperability, and equitable access to these technologies [5]. Patient-reported outcomes, including device comfort and sustained monitoring compliance, are increasingly recognized as determinants of real-world effectiveness [18,19]. Future investigations should examine subgroup effects (such as symptom-driven versus non-symptom-driven indications) and develop signal-quality-aware analytical pipelines that minimize noise artifacts without compromising arrhythmia detection. Despite these advances, several challenges persist. Concerns regarding data quality, false-positive rates, clinical system interoperability, and equitable access require resolution before widespread adoption in routine clinical practice [5].

This study has several limitations. First, the retrospective and observational design introduces the potential for selection bias, as device choice was determined by treating physicians rather than random assignment. This approach, however, reflects routine clinical practice, where device selection is guided by patient symptoms, monitoring objectives, and practical considerations such as expected monitoring duration. In addition, we did not assess how device type may have influenced subsequent patient management. Second, there was an imbalance in sample size between the patch-type and lead-type group. This imbalance reflects real-world clinical practice and did not appear to materially affect the primary outcome. Third, the lead-type group included devices from two different manufacturers, whereas the patch-type group consisted of a single device. Although these lead-type devices share similar technical characteristics and clinical use, unmeasured device-specific differences cannot be completely excluded. Finally, patient-reported outcomes such as comfort, adherence, and device preference were not assessed. These factors are increasingly recognized as important determinants of real-world effectiveness and should be incorporated into future prospective studies, ideally using paired or sequential device comparison designs.

## 5. Conclusions

A direct comparison of patch-type and lead-type wearable ECG monitoring demonstrates that both modalities are clinically feasible and effective. The choice between these devices should be tailored to patient needs, considering monitoring duration, diagnostic accuracy, and noise tolerance. This study contributes practical insights for optimizing wearable ECG technologies in real-world clinical settings. Large-scale prospective studies incorporating patient-centered outcomes across diverse populations are essential to establish the evidence base for these technologies in modern arrhythmia management.

## Figures and Tables

**Table 1:** Baseline characteristics in enrolled patients.

	Total (N = 639)	Lead-Type (N = 173)	Patch-Type (N = 466)	*p*-Value
Age, years	61.7 ± 14.5	61.1 ± 14.8	62.0 ± 14.4	0.504
Sex, male, %	362 (56.7)	93 (53.8)	269 (57.7)	0.418
Medical history, %				
Heart failure	42 (6.6)	10 (5.8)	32 (6.9)	0.754
Hypertension	228 (35.7)	67 (38.7)	161 (34.5)	0.375
DM	78 (12.2)	28 (16.2)	50 (10.7)	0.083
Dyslipidemia	124 (19.4)	33 (19.1)	91 (19.5)	0.987
Stroke/TIA	7 (1.1)	4 (2.3)	3 (0.6)	0.170
CAD	33 (5.2)	12 (6.9)	21 (4.5)	0.302
CKD	17 (2.7)	5 (2.9)	12 (2.6)	1.000

Values in parentheses represent percentages, unless otherwise indicated. CAD: coronary artery disease; CKD: chronic kidney disease; DM: diabetes mellitus; TIA: transient ischemic attack.

**Table 2:** Indication for wearable ECG monitoring.

	Total (N = 639)	Lead-Type (N = 173)	Patch-Type (N = 466)	*p*-Value
Symptom				0.001
Yes, %	505 (79.0)	152 (87.9)	353 (75.8)	
Palpitation	240 (47.5)	70 (46.1)	170 (48.2)	0.736
Dizziness	101 (20.0)	30 (19.7)	71 (20.1)	1.000
Syncope (Pre)	80 (15.8)	24 (15.8)	56 (15.9)	1.000
Chest discomfort	49 (9.7)	16 (10.5)	33 (9.3)	0.805
No, %	134 (21.0)	21 (12.1)	113 (24.2)	
Arrhythmia f/u	62 (46.3)	11 (52.4)	51 (45.1)	0.709
Abnormal ECG	72 (53.7)	10 (47.6)	62 (54.)	0.709

Values in parentheses represent percentages, unless otherwise indicated. ECG: electrocardiogram. f/u: follow-up.

**Table 3:** Baseline characteristics based on symptoms.

	No Symptom (N = 134)	Symptom (N = 505)	*p*-Value
Age, years	59.6 ± 12.8	62.3 ± 14.9	0.040
Sex, male, %	97 (72.4)	265 (52.5)	<0.001
Medical history, %			
Heart failure	14 (10.4)	28 (5.5)	0.066
Hypertension	35 (26.1)	193 (38.2)	0.013
DM	22 (16.4)	56 (11.1)	0.127
Dyslipidemia	39 (29.1)	85 (16.8)	0.002
Stroke/TIA	0 (0.0)	7 (1.4)	0.366
CAD	4 (3.0)	29 (5.7)	0.288
CKD	3 (2.2)	14 (2.8)	0.969

Values in parentheses represent percentages, unless otherwise indicated. Abbreviations are consistent with those presented in Table 1.

**Table 4:** Electrocardiographic parameters.

	Total	Lead-Type	Patch-Type	*p*-Value	Effect Size (Cohen’s d)
	(N = 639)	(N = 173)	(N = 466)		
Monitoring time, h	150.0 [72.0; 168.1]	72.0 [71.7; 72.0]	167.2 [144.9; 168.7]	<0.001	3.97
Total QRS complex, No.	466427.0 [295516.5; 597274.0]	266542.0 [230512.0; 302962.0]	539916.5 [434495.0; 630648.0]	<0.001	0.70
Pause events (>2 s), No.	31.0 [4.0; 532.5]	7.0 [2.0; 53.0]	72.5 [7.0; 777.0]	0.002	0.17
Noise rate, %	6.7 [2.7; 13.8]	2.6 [0.9; 6.7]	7.9 [3.8; 15.0]	<0.001	0.43
AF detection, %	155 (24.3)	43 (24.9)	112 (24.0)	0.911	-

Standardized effect sizes were calculated using Cohen’s d (patch-type minus lead-type). Hedges’ g values were nearly identical and are therefore not shown. Interpretation of effect sizes for pause events should be made cautiously due to the skewed distribution. AF: atrial fibrillation.

## Data Availability

The datasets are available from the corresponding authors upon reasonable request, due to local policies.

## Supplementary Materials

The following supporting information can be downloaded at: https://www.mdpi.com/article/10.3390/jcm15020526/s1, Table S1. Technical characteristics of the wearable electrocardiogram devices; Table S2. Baseline characteristics were categorized by symptom status within each device group.

## Abbreviations

The following abbreviations are used in this manuscript:

AF	atrial fibrillation
CAD	coronary artery disease
CKD	chronic kidney disease
ECG	electrocardiogram
HF	heart failure
ICD	International Classification of Diseases
mHealth	mobile health
TIA	transient ischemic attack
