# Supplementary material for: Comparative Performance of Patch-Type and Lead-Type Wearable Electrocardiogram Devices for Arrhythmia Detection in Routine Clinical Practice
Source: J Clin Med. 2026 Jan 8;15(2):526. doi: 10.3390/jcm15020526 (PMC12842261; doi:10.3390/jcm15020526)
Supplement: Supplementary file 1 [file jcm-15-00526-s001.zip › jcm-4026708-supplementary.pdf]

Table S1. Technical characteristics of the wearable electrocardiogram devices

**Feature**	**Lead-type (Mobicare / S-patch)**	**Patch-type (AT-patch)**
Lead configuration	Lead-based wearable ECG	Leadless adhesive ECG
Frequency bandwidth	0.05-55 Hz /0.05-40Hz	0.05-40 Hz
Sampling rate	256 Hz	250 Hz
Adhesive	Replaceable electrodes	Integrated patch
Battery life	Up to 3 days	Up to 14 days
Battery	Built-in battery	Replaceable coin battery
Reattachment	Possible	Not recommended

ECG: electrocardiogram

Table S2. Baseline characteristics were categorized by symptom status within each device group.

	Lead-type			Patch-type		
	No Symptom (N=21)	Symptom (N=152)	P-value	No Symptom (N=113)	Symptom (N=353)	P-value
Age, years	62.9 ± 13.9	60.8 ± 14.9	0.550	59.0 ± 12.5	62.0 ± 14.4	0.007
Sex, male, %	13 (61.9)	80 (52.6)	0.572	84 (74.3)	185 (52.4)	<0.001
Medical history. %						
Heart failure	1 (4.8)	9 (5.9)	1.000	13 (11.5)	19 (5.4)	0.043
Hypertension	7 (33.3)	60 (39.5)	0.762	28 (24.8)	133 (37.7)	0.017
DM	7 (33.3)	21 (13.8)	0.050	15 (13.3)	35 (9.9)	0.407
Dyslipidemia	5 (23.8)	28 (18.4)	0.770	34 (30.1)	57 (16.1)	0.002
Stroke/TIA	0 (0.0)	4 (2.6)	1.000	0 (0.0)	3 (0.8)	0.759
CAD	1 (4.8)	11 (7.2)	1.000	3 (2.7)	18 (5.1)	0.407
CKD	1 (4.8)	4 (2.6)	1.000	2 (1.8)	10 (2.8)	0.780

Values in parentheses represent percentages, unless otherwise indicated. Abbreviations are consistent with those presented in Table 1.
